# Outcome Analysis of Congenital Diaphragmatic Hernia Cohort before and after Implementation of Standardized Protocol in a Tertiary Neonatal Unit

**DOI:** 10.1055/s-0037-1606221

**Published:** 2017-08-24

**Authors:** Amitava Sur, Adjemoke Awoseliya, Alok Sharma

**Affiliations:** 1 Southampton University Hospitals NHS Trust, Southampton, Hampshire, United Kingdom

**Keywords:** congenital diaphragmatic hernia, pneumothorax, standardized guidelines

## Abstract

Despite evolving evidence and increased understanding, there is a strong argument that best outcomes in managing congenital diaphragmatic hernia (CDH) patients are achieved in centers which have a high admission rate of such patients and follow standardized operating protocols of management. Pneumothorax and air leak syndromes are one of the main causes of pre- and postoperative morbidity in these patients and experts believe that delivery room sedation and gentle ventilation strategies can minimize this. We observed a significant drop in incidence of pneumothorax and reduction of mortality following implementation of standardized guidelines at the neonatal unit at Southampton which is a tertiary-level neonatal care in the southern United Kingdom and a regional referral center for CDH patients.

Congenital diaphragmatic hernia (CDH) remains one of the most challenging therapeutic considerations in neonatal medicine requiring close liaison between multidisciplinary care groups. With an incidence of 1 in 3,000 live births, the postnatal mortality remains between 50 and 70% despite advances in treatment modalities. 1 A recent report from the Canadian Neonatal Research Network indicates better than predicted survival in network centers, with significant improvement in the higher mortality odds strata, and 90% survival in the three centers that cared for at least 12 infants over a 22-month period. The report suggests that survival is best achieved by caring for CDH infants at centers that have developed a standardized approach to care, with adequate experience in CDH management. 2 Ventilation strategies in CDH care have evolved from the era of hyperventilation and alkalosis to “gentle ventilation” and permissive hypercapnia. Even with the gentler strategies, pneumothoraces remain one of the most frequent and critical complications of CDH management with an incidence of 18 to 36% reported in literature. 3 4 5 Immediate intubation, delivery room sedation, and paralysis and the early surfactant have been reported as practices adopted by neonatal units. 6 7 In a large cohort of 510 patients reported by a Japanese tertiary center, 13.5% developed pneumothorax and were associated with a significantly high mortality (55%). 8 The practices of management of CDH vary widely even among regions, with extracorporeal membrane oxygenation (ECMO) centers having a better overall antenatal diagnosis and initial management. In an article describing the Western Canada experience, the antenatal diagnosis was found to be 46% and the practice of delivery room sedation and paralysis varying from 55% in ECMO centers to as low as 26% in others. 2

Although the quality of life assessments have become a standard practice in high-risk populations, data in CDH patients is not extensive. Neurodevelopment and neurofunctional morbidities are the major problems in CDH survivors, 9 with data showing 25 to 50% of survivors will have at least mild delay, the risk being higher in those subjected to ECMO. 10 11 12 Data on preschool assessment in this cohort are sparse.

The current study was a quality improvement project in the management of CDH at the Princess Anne Hospital, Southampton, United Kingdom. This is a tertiary neonatal unit with 24 hours pediatric surgery and pediatric cardiology services. The unit experiences five to seven CDH deliveries/year on an average, being the regional referral center for CDH deliveries. The center does not presently have ECMO facilities, but the pediatric intensive care performs bridging ECMO for a referral to ECMO centers. The standardized operating protocol (SOP) for delivery room management of CDH was formulated in December 2012 and is given in the Supplementary Material (online-only). There exists no current dedicated neurodevelopment follow-up clinic for CDH patients. So we decided to use a prevalidated parent questionnaire 11 via a telephone survey to try and obtain a crude assessment of the motor and language skills of these patients.

## Objectives

Primary objective: This study compares outcome analysis of pre- and postimplementation of SOP with regards to mortality and incidence of pneumothorax.

Secondary objectives: (1) To analyze the incidence of morbidities such as chronic lung disease and incidence of home ventilation and association of pneumothorax with these in this cohort. (2) Telephonic survey of parents with a standardized questionnaire for a crude assessment of developmental status and chronic morbidities among the discharged survivors of the cohort.

## Materials and Methods

A retrospective analysis was conducted for all patient discharge diagnosis data of CDH from 2007 to 2016. Databases accessed were surgical records, e-documents, and automated discharge summaries of patients. Data used for indicators of morbidity were:

1. Missed antenatal diagnosis
2. Liver up on first scan/X-ray
3. Nasogastric tube insertion at delivery/first X-ray
4. 10th day of surgery
5. Hospital length of stay
6. Mode of ventilation
7. Ventilation days
8. ECMO
9. Inhaled nitric oxide (iNO)
10. Inotrope days/number of inotropes used
11. Chronic lung disease
12. Discharge on home oxygen

All data were collected and stored in password-protected hospital computer drive in an anonymous format.

For comparative analysis, the data were categorized into two epochs: 2007 to 2012 and 2013 to 2016.

Statistical analysis was done using SPSS version 23.0 (SPSS Inc.). Descriptive statistics and simple frequency distribution charts were used to describe baseline demographics. Pre- and postcomparisons of incidences of mortality and pneumothorax were done using Fisher's exact test. Binary stepwise logistic regression analysis was undertaken to analyze predictors of outcome.

For the developmental follow-up, parents were telephoned, and asked the questions, if they consented, and data were stored anonymously in an online software and questionnaire tool.

## Results

The total number of patients admitted with the diagnosis of CDH in the specified time period was 69 (44 in the first epoch and 25 in the second epoch). The baseline demographics were comparable among the two cohorts and are described in Table 1 .

**Table 1:** Baseline demographics

	Total	Epoch 1	Epoch 2
Antenatally diagnosed	42/69	31/44	11/25
Mean GA (wk)	37.9 (IQR: 37, 39)	37.6	38.4
Mean BW (g)	3,045 (IQR: 2,641, 3,426)	3,018	3,090
Mean discharge day	20.7 (IQR: 9, 33)	17.52	23.5
Mean ventilation days	9.5 (IQR: 2, 16)	6.3	12.7
Mean day of surgery	3.2 (IQR: 2, 4)	2.7	4.6
Surgical repair	52 (76.9%)	27/44	25/25

Abbreviations: BW, birth weight; GA, gestational age; IQR, interquartile range.

**Fig. 1:**
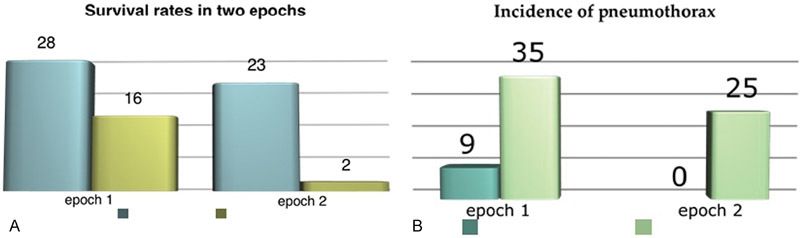
( **A** ) The survival rates in two epochs. ( **B** ) The incidence of pneumothorax. The incidence of pneumothorax in two epochs.

The primary outcome parameters showed a significant improvement. The mortality rate dropped from 16/44 patients (36.36%) in the first epoch to 2/25 (9.5%), *p* = 0.01. The incidence of pneumothorax was also significantly decreased from 9/44 (29%) to none reported in the last epoch (0/25), *p* = 0.02 ( Fig. 1A, B ).

Regression analysis showed pneumothorax to be significantly associated with mortality, *p* = 0.045 (95% confidence interval: −1.05, 53.3) when adjusted with other determinants, such as “time of diagnosis-antenatal/postnatal,” “left-sided or right-sided defect” and “liver in thoracic cavity or liver down.”

Chronic lung disease is a major cause of respiratory morbidity in these patients, and the most severely affected ones are often discharged on home oxygen. In our cohort, eight patients (11.6%) were discharged home on oxygen. Of these patients, five had a radiological diagnosis of pneumothorax in the preoperative period. So pneumothorax was also significantly associated with discharge on home oxygen, *p* = 0.02.

Comparative analysis of primary parameters of morbidity between the two epochs is detailed in Table 2 . It was observed that the patients in epoch 2 were diagnosed to have significantly more incidences of liver visualized in the thoracic cavity in the antenatal scan ( *p* = 0.013), had more ECMO referrals ( *p* = 0.002), and had more incidences where sildenafil ( *p* = 0.029) and prostaglandin was used ( *p* = 0.01).

**Table 2:** Prognostic indicators of severity

Morbidity parameters	Epoch 1	Epoch 2	*p* Value
Liver up on scan	5/ 44	10/25	0.013
Mean peak inspiratory pressures	22	22.8	
Mode of ventilation (HFOV)	23/44	11/25	0.61
PPHN (Echo evidence)	27/44	14/25	0.79
> 1 inotrope used	12/44	10/25	0.29
Sildenafil used	3/44	7/25	0.029
iNO used	26/44	12/25	0.45
Prostin used	0/44	4/25	0.01
ECMO referral: 8 (3 performed, 2 survived)	1/44	7/25	0.002
Chronic lung disease	5/44	5/25	0.47
Discharged on home O _2_	6/44	2/25	0.7

Abbreviations: ECMO, extracorporeal membrane oxygenation; HFOV, high-frequency oscillatory ventilation; iNO, inhaled nitric oxide; PPHN, persistent pulmonary hypertension of the newborn.

### Results of Parent Questionnaire

**Fig. 2:**
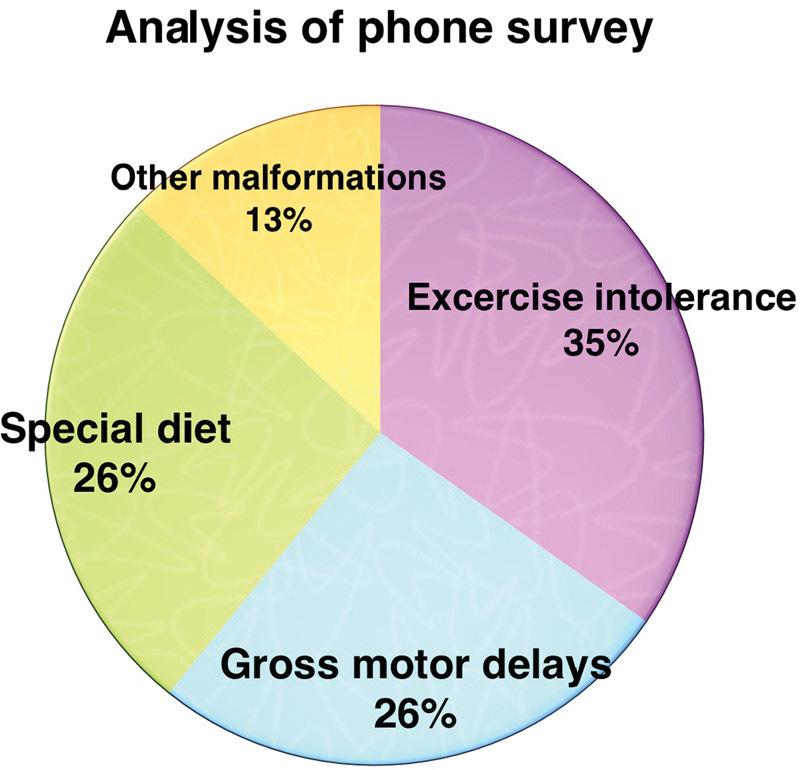
Frequency of common complications/sequele in discharged patients as indicated by phone survey.

Parents of 23 patients responded to our survey. Each telephonic survey took approximately 10 to 15 minutes. Gross motor delays (delay/inability to sit/walk at an appropriate age) were present in 26% patients. As many as 35% had some form of exercise intolerance. Oromotor problems are also a recognized complication of the severely affected patients. We found that 26% were on a special diet with four patients still on home nasogastric/percutaneous endoscopic gastrostomy (PEG) feeds ( Fig. 2 ).

## Discussion

The recent update on CDH management published by the Euro Consortium 13 has emphasized that an experienced tertiary center with a high volume of CDH deliveries (>6/y) is the optimal environment for management of antenatally diagnosed cases. Being the regional referral center, our unit has gained a considerable experience as a team (including neonatology and pediatric surgical services) in standardizing management. Regarding delivery room management, although the Euro consortium has advised against routine neuromuscular blockade after delivery, this has remained a standard of care in our unit as one of the measures to prevent pneumothorax. With gentle ventilation and opting for high-frequency oscillation early if peak inspiratory pressures exceeded 25 cm H _2_ O, we have managed to minimize the incidence of pneumothorax. From 2014 onwards the judicious use of prostaglandin E1to keep the ductus open guided by echocardiography, has also been adapted as a standard of practice to help off load the right ventricle. This approach has also been highlighted in the recent guidelines. 14 The use of targeted echocardiography to manage hemodynamic instability is of paramount importance, especially in the first 24 hours, helping us to decide on the choice of inotropes and pulmonary pressures. The round the clock availability of on-site pediatric cardiology services help us improve management of these often sick patients. The patients who require ECMO services are referred to centers which deliver ECMO in London or Glasgow, United Kingdom.

The result data provided in Table 2 indicate a higher proportion of infants on the severe spectrum in epoch 2. The fact that there has been a significant reduction in mortality and incidence of pneumothorax in this epoch supports the evidence behind standardization.

These infants require a dedicated neurodevelopmental follow-up service, and our aim is to work toward setting up one. At the moment they are followed up individually by the neonatal team and surgical team as outpatients. After our primary experience through the telephonic survey, a developmental clinic is being planned to try to address these problems.

This study, in essence, is a quality improvement analysis aiming to report significant improvement in mortality and morbidity after implementation of standardized guidelines for management. The study is limited by relatively small cohort and often incomplete or missing clinical data due to its retrospective nature. But it addresses a significant complication of CDH patients, namely, pneumothorax and its potential burden on outcomes and also emphasizes the value of standardized care in the management of such complex patients.
