# Supplementary material for: Outcome Analysis of Congenital Diaphragmatic Hernia Cohort before and after Implementation of Standardized Protocol in a Tertiary Neonatal Unit
Source: Surg J (N Y). 2017 Aug 24;3(3):e139–42. doi: 10.1055/s-0037-1606221 (PMC5570593; doi:10.1055/s-0037-1606221)
Supplement: Supplementary file 1 — Supplementary Material [file 10-1055-s-0037-1606221_s1600106oa.pdf]

University Hospital Southampton NHS
NHS Foundation Trust

# Postnatal Management of Congenital Diaphragmatic Hernia

Version: 5

| | |
|---|---|
| Date Issued: | December 2015 |
| Review Date: | December 2018 |
| Document Type: | Clinical Guideline or Guideline<br>PDF |


| Contents | | Page |
|---|---|---|
| Paragraph | Executive Summary | 2 |
| 1 | Background | 3 |
| 2 | Guideline-Preparation prior to delivery | 4 |
| 3 | Guideline-Delivery room management and stabilisation | 5-6 |
| 4 | Guideline NICU Management | 7-14 |
| 5 | Guideline Postoperative Management | 15 |
| 6 | Prognosis & Follow Up | 16 |
| 7 | Monitoring Compliance and Effectiveness | 16 |
| 8 | References | 17-20 |

| Appendices | | Page |
|---|---|---|
| Appendix 1A | Equipment for Airway & Access in Delivery Suite | 21 |
| Appendix 1B | Drugs for sedation paralysis | 22 |
| Appendix 2 | Imminent Delivery of a baby with CDH | 23 |
| Appendix 3 | CDH Management Pathway (Flowchart) | 24 |


## Document Status

**This is a controlled document. Whilst this document may be printed, the electronic version posted on the intranet is the controlled copy. Any printed copies of this document are not controlled.**
**As a controlled document, this document should not be saved onto local or network drives but should always be accessed from the intranet.**

# Executive Summary

## SUMMARY BOX AND LEVELS OF EVIDENCE[1-31]

| | |
|---|---|
| **After delivery, the infant should be intubated immediately without bag and mask ventilation** | D |
| **Aim for preductal saturations between 85-95%** | D |
| **Avoid PIP of>25 where possible** | D |
| **Consider HFOV if conventional ventilation fails** | D |
| **PPHN should be treated with NO** | D |
| **ECMO may decrease mortality** | D |
| **Surgical repair should be delayed until physiologically stable** | D |

## NATIONAL RECOMMENDATIONS FROM THE CONFIDENTIAL ENQUIRY (MBBRACE)

### Recommendations

- Commissioners, service planners and policy makers should consider the establishment of clinical networks that manage the care of babies with CDH. This would include focusing the acute care of these babies on a limited number of centres in order to facilitate the development of:
  - Multidisciplinary teams capable of providing care focussed on both the mother and the baby;
  - A collaborative approach to R&D allowing care to become more evidence based;
  - Sub-specialty expertise in the management of all aspects of care;
  - An agreed 'national information sheet' to provide consistency of information about the condition;
  - Consistent counselling throughout the care pathway;
  - Quality improvement and the sharing of best practice;
  - Collaboration with the proposed national congenital anomalies register to ensure complete case ascertainment;
  - Consensus on the optimal management of the care and treatment for babies diagnosed with CDH and the management of late termination of pregnancy in this group of women.
- Commissioners, service planners and policy makers should consider the development of a service specification for CDH to ensure the service becomes focussed on the needs of the family with, for example:
  - Access to psychological support where applicable;
  - Adequate follow-up arrangements.
  - Organisation of care during the antenatal period in a way that minimises the need for travel;
- There is a continuing need to highlight the importance of documentation to the whole multidisciplinary team involved in the care of women and babies following a diagnosis of CDH.

# 1. Background

Congenital diaphragmatic hernia (CDH) occurs in approximately 1 in 3000–4000 live births, and is associated with a high overall mortality and a high rate of morbidity amongst survivors. Estimates of the number of cases in the UK vary but it is likely that there are between 200 and 300 new cases annually. Of these up to 70% are likely to be diagnosed antenatally as part of routine screening. Up to a third of all UK cases end in either a spontaneous loss during the pregnancy or an elective termination. A national confidential inquiry on the management of neonates with CDH delivered in 2009-10 identified enormous variation across the UK in the postnatal management of neonates with CDH.[1] The following pathway covers a standard approach based on the recommendations on good clinical practice made by the Topic Expert Group of the committee set for the national confidential enquiry and current available literature. This guideline covers the management of the baby with a congenital diaphragmatic hernia after delivery. [1, 12] This guideline does not cover the antenatal pathway. For detailed further information regarding the antenatal management of CDH and information such as lung head ratios and issues such as FETO the following references are available. [3-8]

The key tenets of this guideline are establishing uniformity in practice, and clear pathways for management and escalation in the management of neonates with CDH. It must be recognised that with the best of knowledge and skills human factors play a critical role in management. For some background please click here to view the report and recommendations of the National Confidential Enquiry into Congenital Diaphragmatic Hernia conducted by MBBRACE-UK.

An overview is provided in Appendix 1, 2 & 3to summarise the important tenets of management.

# 2. Guideline- Preparation prior to delivery

## General Principles

A multidisciplinary team should be allocated to attending delivery to include a senior member of staff (consultant). Preparation should be made before hand and if time allows, the care of the baby should be discussed with the parents. Tasks should be allocated to members of resuscitation team prior to baby's delivery identifying responsibility for airway management, access and medication delivery. Resuscitation equipment should be prepared beforehand by both medical and nursing team. The labour room resuscitaire should also be thoroughly checked. Below is a list of equipment for the delivery room. Note that surfactant should not be drawn up routinely. This should only be prepared for premature babies who would normally receive this for surfactant deficiency. **Consider discussion with PICU at this stage if considered to be high risk and may require ECMO (to facitilitate their resource planning).**

## List of equipments for delivery room
## (Including but not limited to Appendix 1A &1B)

- Printed copy of management flow chart
- Pre-cut endotracheal tubes with introducers
- Net-elast hat and ties
- Laryngoscope and blades
- Suction catheter including a Yankuer sucker
- 24G Cannulas
- Single lumen UVC with cord tie and blade (For failure of cannulation)
- T- piece flushed through and attached to syringe
- Aliquots of normal saline 10mls/kg (depending on estimated weight of baby)
- Nasogastric tube with purple syringes
- 2 doses of sedation, preferably Fentanyl.
- 2 doses of muscle relaxant, also prescribed according to estimated weight
- 0.9% saline flushes, two 5ml syringes as flush.
- Saturation monitor

Note that resuscitation where possible should be under direction of a consultant or senior personnel experienced in management of such babies.

An estimated weight using the 50$^{th}$ centile for gestation can be used for drug calculation.

**Where delivery of a baby with a CDH is suspected preparation of a bed space on the neonatal unit with the appropriate ventilator and delivery of Nitric Oxide must be considered prior to delivery if time allows. The HFOV (Sensormedics) may be needed.**

## 3. Guideline-Delivery room management and stabilisation Standardised Approach(See figure 1 and Training Video )

**Personnel**-Resuscitation should be led by a consultant. Ideally, at least two experienced resuscitators and a nurse should be in attendance at the delivery. One of which should be a senior neonatologist (consultant when possible), together with an expert assistant.

**Oxygen**-Use 100% oxygen for resuscitation and ventilation [12].

**Intubate**- Intubate immediately without bag and mask ventilation. Ventilation by bag and mask may cause distension of the stomach and must be avoided as it may limit expansion of the hypoplastic lung. Rapid intubation reduces a possible risk of pulmonary hypertension due to prolonged acidosis and hypoxia which may result from delayed intubation.
Do not delay intubation whilst obtaining intravenous access [12].

**Pressure**-Aim for the minimum PIP that enables adequate chest expansion and ventilation. Ideally should be≤25 cm of H20 with a PEEP 5 but clinical judgement is needed here and higher pressures may be needed [12].

**Monitoring**-Attach a saturation probe to the right hand and aim for sats of 80-95% by 15 minutes of age where possible [12].

**Decompress the Stomach**-An orogastric or nasogastric tube (large bore 8-10) should be placed to decompress the bowel and left on free drainage. This will help to prevent bowel distension and any further lung compression.

**Access**-A peripheral venous line should be inserted for sedation and paralysis in delivery suite. In an emergency a UVC may become necessary but where possible umbilical lines should be inserted in a controlled environment i.e. NNU

**Sedation and Paralysis**-As soon as venous access is established, give a dose of Fentanyl (4microgram/kg) and a paralytic agent. This helps prevent the physiological response to awake intubation which may be uncomfortable raising intracranial pressure, pulmonary pressure, causing bradycardia. Paralysis along with sedation is recommended to ensure the baby is not fighting. . In the absence of IV access, I/M Suxamethonium can be given at a dose of 4milligrams/kg.

**Cardiovascular Assessment**- A clinical evaluation of the cardiovascular status is necessary. 10mls/kg of crystalloid can be given 1-2 times at clinical discretion.

**Surfactant**-Delivery room surfactant for these babies is not recommended.

**Temperature**- Maintain normothermia [12].

# Figure 1
# Delivery room management

**Intubate**
**5 Inflation breaths (each 2-3sec) at <25 where appropriate (30cmH2O max)**
**Confirm chest wall movement and auscultate**
**Secure ETT**

*Resuscitator 1*
*Helper 1*

- **Aim to reduce PIP to 25cmH2O**
  **Continue ventilation breaths**
- **Ensure good chest movements**
  **Keep HR>100**
- **If HR <100, reassess ABC**
  **Follow NLS algorithm**
- **If poor response, ensure:**
  **-In 100% O2**
  **-ETT in correct position**
  **-Consider increasing PIP**
  **-Consider pneumothorax**

*Resuscitator 2*
*Helper 2*

- **Pass large bore oro/nasogastric tube**
  **Aspirate and leave on free drainage**
- **Obtain IV access**
- **Administer sedation and flush**
- **Give muscle relaxant if required**
- **Prepare transport incubator and update parents**

**Transfer baby to NICU**
**Please inform PICU consultant and surgical team that baby has delivered**

## 4. Guideline-NICU Management

Management should be led by the service/on call neonatal consultant**.**

### Ventilation management-General Principles

1) The optimal initial ventilation mode for newborns with CDH varies. There is accumulated evidence that ventilator induced lung injury may have a significant negative impact on outcome in newborns with CDH [13-16]. There is also a risk of precipitating a pneumothorax through use of high pressure ventilation. Having said that persistent hypoxia can result in intractable PPHN. Permissive hypercapnia and gentle ventilation in neonates with CDH has been reported to increase survival [17-20]. Early postnatal ventilator management would be a balance between achieving adequate oxygenation and not driving the lung too hard.

2) **Lung Protective Strategy**-The European CDH Consortium Consensus recommends adapting treatment to target a preductal saturation level between **85% and 95%** and a Postductal saturation of >70% where possible. This is in keeping with a lung protective strategy outlined in the confidential enquiry into CDH. This will often need high oxygen delivery and even Nitric Oxide and after stabilisation, Fio2 should be decreased if preductal saturations are persistently >95% [1, 12].

3) **Consideration should be given to the following**
Aim for Paco2 of between 45 and 60mmHg (6-8Kpa)
Try to limit PIP to 25mmHg or less, and PEEP to 5cm H20.
Consider HFOV early in neonates needing higher pressures.
Ensure adequate sedation and paralysis with intravenous Morphine (and Midazolam if necessary). Continue paralysis after delivery room management.
Administer a bolus of a muscle relaxant if breathing is asynchronous or ventilation is proving difficult despite the above measures.
A chest X ray should be done as soon as possible after delivery and stabilisation to assess recruitment.
Ensure blood gas analysis occurs regularly and ideally within 1 hour of a significant change to ventilator settings.
Document all changes on the gas chart along with the oxygenation index (OI, OI=mean airway pressure x FiO2 x 100/PaO2. [12].

4**) PPHN**- Anticipate PPHN. Early echocardiography and treatment are very important to guide management here. Indications for Nitric Oxide are a high oxygen requirement, difference between pre and post ductal saturations and lack of oxygenation on high FIO2 and inotropes. (See PPHN section)

5**) Surfactant**-The use of surfactant has not shown to improve survival. It can be considered at consultant discretion on a case by case basis or where RDS is thought to be an additional pathology as may be the case in a preterm baby with CDH [12].


Disclaimer: It is your responsibility to check against StaffNet that this printout is the most recent issue of this document

# Ventilation-Which Mode To Use?

There are no data to show the superiority of either conventional or high frequency oscillation as the primary ventilation support. Most babies will start with conventional ventilation [21, 22].

## Conventional

It is worthwhile trying conventional ventilation, however if a PIP of over 25cm H20 is necessary to achieve Pco2 and saturation levels within the target range despite being in oxygen, consider (HFOV)

## High frequency Oscillatory Ventilation (HFOV)

The physiological rationale for use of HFOV derives from its ability to preserve end-expiratory lung volume while avoiding over distension, and therefore lung injury at end-inspiration. HFOV may improve gas exchange, promote uniform lung inflation, reduce barotrauma, and decrease the presence of inflammatory mediators [13, 14, 15, 22].

The indications for HFOV are not clearly defined but it is mostly used as rescue therapy in patients with persisting hypoxemia and hypercapnia on conventional ventilation. [12]

Initial setting; mean airway pressure 2 above conventional MAP setting, frequency 8-10Hz, DP 30-50cmH20 depending on chest wall vibration to produce visible wobble [12].

The Mean airway pressure should be adjusted to have an adequate expansion of the lungs but avoid over inflation. Over inflation can compromise venous return to the heart and function. This can make PPHN worse.

A chest x-ray should be performed within half an hour to confirm the lungs are not overinflated, as defined by a contra lateral lung expansion such that more than 8 posterior ribs are visible above the diaphragm.

It must be remembered that for big term neonates the Sensormedics may be needed to deliver HFOV. This takes time to set up and if delivery of a baby with a CDH is expected this and the Nitric Oxide should be set up next to the baby.


Disclaimer: It is your responsibility to check against StaffNet that this printout is the most recent issue of this document

# Hemodynamic management

## Assessment & Access

Where possible monitor BP through an umbilical arterial catheter or peripheral arterial line (preferably right radial arterial line as it reflects a preductal po2). A central line (double lumen UVC) should be inserted in the NICU. Aim to achieve appropriate end organ perfusion maintaining a gestation appropriate blood pressure and a blood pressure above a mean of 40-45mm of Hg in the term baby without PPHN. Monitor heart rate, capillary refill, urine output and lactate levels.

An echo is usually warranted in the 1$^{st}$ 24 hours.
A stable cardiovascular status is indicated by a combination of the following
-Heart range is within normal range
-Capillary refill is below 2-3 seconds
-Urine output over 1.0ml/kg/hr.
-Lactate less than 3mmol/l
-Normal volume pulses

## Management

A key finding of the confidential enquiry into CDH management was the use of fluid without an assessment of the risks of fluid overload.

**Echocardiography**-If symptoms of poor perfusion and/or blood pressure below the normal for gestational age occur request an Echocardiography assessment to differentiate hypovolaemic from cardiogenic shock. It also helps look at ventricular function and diagnose PPHN. The management will differ depending on the underlying problem. Please be specific with the paediatric cardiologist about the question you are asking and ask for senior review by a consultant paediatric cardiologist where indicated to guide management.

Consider giving 10-20mls/kg of 0.9% saline cautiously if no volume has been given previously to help preload. Excessive fluid resuscitation with myocardial dysfunction can worsen the situation. If greater than 20ml/kg volume are required consider early inotropes, starting with 10mcg/kg/min of dopamine.  Assess for PPHN requiring nitric oxide and vasopressors.  Echo forms part of this assessment but do not delay starting nitric oxide and inotropes whilst waiting for an echo.  Ensure the consultant is aware of any escalation of therapy that is required.

Hydrocortisone may be used for treatment of hypotension after failure of conventional treament and there is inotrope resistant hypotension.

Discuss with PICU if early if there are signs of myocardial dysfunction, cardiogenic shock or severe PPHN as the baby may require consideration for ECMO.

# Management of Pulmonary Hypertension

The optimal treatment of CDH associated pulmonary hypertension is one of the major challenges. Support of the systemic circulation and acceptance of variable degrees of right to left shunting are cornerstones of the therapeutic approach. With severe PPHN it may be required to optimise the right to left shunt in order to off load the Right ventricle and maintain systemic perfusion. Serial echocardiograms by an experienced cardiologist may be required. Interpretation of the echo can be difficult and a consultant cardiology opinion must be sought early if the patient is unstable.

Pulmonary hypertension may be indicated by difficulty in oxygenation, a significant (>10%) difference between Pre and Postductal saturations, or signs of poor systemic perfusion in the face of difficult ventilation. It should also be considered if the Oxygenation Index is over 20. If the ductus arteriosus is closed there will be no pre / post ductal saturation difference, even in the face of severe pulmonary hypertension. Prostaglandin may be required to augment the circulation in this situation if the patient is in cardiogenic shock. Treatment of pulmonary hypertension may require multiple strategies started simultaneously.

## 1) Optimise Physiology

a) Metabolic Acidosis-It impairs myocardial function and results in pulmonary vasoconstriction. Treat with sodium bicarbonate or THAM

b) Transfusion can help optimise the oxygen carrying capacity

c) Optimise ionised Calcium and Serum Magnesium in support of blood pressure

d) Maintain axillary temperature between 36.6-37.5C. Avoid **hypothermia** and hyperthermia.

e) Good sedation and analgesia are important as pain can make pulmonary hypertension worse. Pain also causes tachycardia which impairs ventricular filling.

f) The use of a high mean airway pressure or PEEP can impair venous return to the right heart making PPHN worse.

g) Review your chest Xray to look at the heart size and lung expansion.

## 2) BP

a) Volume-10-20mls/kg of saline or HAS can be used but avoid overfilling as this can make ventricular function worse.

b) In PPHN It may well be that a higher systemic BP is needed to prevent right to left shunting based on echo assessment to treat PPHN. In addition there may be problems with right ventricular dysfunction.

c) The choice of inotropes is at consultant discretion & may need close liaison with the paediatric cardiology, PICU team. Dopamine or Noradrenaline may be suitable depending on gestation.

d) Consider use of Prostaglandin E1 early if there is right ventricular dysfunction with PPHN and a restrictive PDA.

e) Consider use of Hydrocortisone where there is poor response to inotropes.

f) The use of Milrinone in addition to Noradrenaline in right ventricular dysfunction can help offload the right ventricle.


Disclaimer: It is your responsibility to check against StaffNet that this printout is the most recent issue of this document

## Nitric Oxide & Vasodilators

1. Consider use of inhaled Nitric Oxide (20ppm) early in care. In the absence of an echo pulmonary hypertension may be indicated by d**ifficulty in oxygenation with a pre and post ductal saturation difference of 10%.** This should be tried for at least one hour. Although a randomised controlled trial did not demonstrate a beneficial effect for inhaled nitric oxide [23], the immediate short term improvement in oxygenation may be beneficial as a bridging period to ECMO.

2. Consider IV prostaglandin E1 in cases of suprasystemic pulmonary arterial pressure and right to left shunting through the foramen ovale on echo especially if you have right ventricular dysfunction and a restrictive PDA.

3. IV Magnesium is a pulmonary vasodilator and can be administered in cases of refractory pulmonary hypertension. It can make things worse by causing systemic hypotension through vasodilatation and may need escalation of vasopressors. It's use should be discussed with the consultant and closely monitored.

4. Sildenafil and other pulmonary vasodilators (Prostacyclin) for PPHN have been used on a case by case basis to treat PPHN. There is no evidence that these help in the acute phase in infants with CDH however they may benefit a baby with suprasystemic pulmonary hypertension and after consultation with a cardiologist use on a case by case basis may be warranted. [12]

## Sedation and Analgesia

**Steps (12)**

Keep the environment quiet! Avoid handling where possible. Preoxygenate prior to handling where possible by increasing FIO2 by 20% if appropriate.

Continuous morphine infusion is the drug of choice in our unit. Morphine boluses may be required. Titrate according to clinical assessment, heart rate and blood pressure as needed.Beware of hypotension and be prepared for inotropic support with increasing doses of sedation.

Midazolam may be used as a suitable adjunct where asynchrony with ventilation and agitation causing hypoxaemia are problems.

Rectal Chloral hydrate is also an option when NBM or orally when fed.

The use of paralysis in clinical practice is almost the norm because of significant asynchrony, difficulty in obtaining adequate sedation resulting in compromised ventilation and significant PPHN. It must be remembered however that neuromuscular blocking is associated with side effects like hypoxaemia, worsening oedema, 3rd spacing, and inability to bring up secretions. Consider using and weaning infusions to the lightest dose necessary to aid sedation and stopping if not needed.


Disclaimer: It is your responsibility to check against StaffNet that this printout is the most recent issue of this document

# Extracorporeal Membrane Oxygenation (ECMO)

ECMO has been reported to improve survival in infants with CDH [24, 25]. The utilisation of ECMO ranges from 15 to 40% [26]. The overall survival of CDH following ECMO is 51% [27]. In many centres, ECMO is considered in infants with CDH if there is evidence of an adequate amount of lung parenchyma suggested by a period of adequate preductal oxygenation and/or ventilation. The national confidential enquiry recommends that in cases where the baby could be adequately stabilised and the degree of pulmonary hypoplasia was felt to be compatible with long term survival ECMO should have been considered. The case should be discussed early with an ECMO Centre. Following discussion with an ECMO Centre, the case for/against ECMO should have been discussed with parents. The reasons for its use, its limitations and why it was/was not advisable for the treatment of their baby should have been documented. It must however be remembered that wider issues might preclude ECMO. Other co morbid conditions need to be considered before referral for ECMO. These include prematurity, presence of significant Intraventricular haemorrhage, lethal malformations and congenital anomalies and syndromic diagnosis with poor prognosis. ECMO is only worth considering if the baby has lungs which are compatible with survival. In babies with severe pulmonary hypoplasia or where there are other congenital abnormalities ECMO does not play a useful role. If the baby has never had a preductal PaO2 above 4-5kPa and ventilation has been difficult from the beginning despite maximal treatment then ECMO may be inappropriate. Please note that despite use of ECMO, mortality remains high. ***Babies are to be discussed for ECMO after careful multidisciplinary discussion with the PICU and surgical consultants.*** It is essential that if a patient is not responding to treatment, or deteriorating, then ECMO is considered early and the patient is discussed with the UHS PICU consultant in a timely manner, as they may be in a position to offer help with stabilisation, or help facilitate referral and transfer for ECMO. Patients do not need to be transferred to PICU if they have been accepted for ECMO and can be retrieved in a timely manner by the ECMO centre.

In extreme cases ECMO may need to be initiated at UHS, on the PICU, as a temporary stabilising measure. This will only occur if the patient has been accepted for ECMO by a lead centre but is too unstable to wait for retrieval, or there are no UK beds available and it is agreed with the lead centre that the patient is placed on ECMO in UHS whilst awaiting a bed.

## Criteria for ECMO referral [12]

Indicators that should prompt a referral and discussion are as below (not limited to these criteria individually)-

- OI ≥ 40 or inability to maintain preductal saturation of >85% or postductal of >70%
- OI >20 consistently, with signs of cardiogenic shock requiring high dose inotropic support.
- Increased Paco2 with pH<7.15 despite optimising ventilator management
- Systemic hypotension resistant to fluid and inotropic therapy with urine output of <0.5ml/kg/hr. for at least 12 to 24 hours
- Inadequate oxygen delivery with metabolic acidosis as measured by lactate >/- 5 and pH<7.15
- Rapid deterioration or severe ventricular dysfunction [28]
- Severe air leaks unresponsive to other therapies [28]
- No contraindication to ECMO (see above)

## Fluid management and Parenteral Nutrition

- Fluid restriction (40-60mls/kg/day) should be considered in the first 24 hours. Strict input and output chart should be kept.
- The use of paralysis is likely to make these neonates oedematous.
- Total parenteral nutrition (TPN) should be started within 24 hours once suitable central access has been obtained. These babies are not likely to be fed until after surgery.
- Monitor electrolytes daily to start with. If stable, they can be checked with TPN bag changes.
- Consider diuretics where appropriate in case of a positive fluid balance. Aim for diuresis of 1-2ml/kg/hr. A low albumin may necessitate the need of 20% Albumin on a case by case basis. It is important to monitor renal function closely in such situations.


Disclaimer: It is your responsibility to check against StaffNet that this printout is the most recent issue of this document

# Timing of Surgery

Consensus exists that repair of the diaphragmatic defect should be done as a semi-elective procedure once the baby has stabilised. Physiological stabilisation [11] has been defined as

- Mean arterial blood pressure normal for gestational age.
- Preductal saturation level of 85-95% in $FiO_2$ <50%.
- Lactate<3mmol/L.
- Urine output >2ml/kg/hr.

A key area is making a judgement that pulmonary pressures are stable and that there is good myocardial function. The decision regarding operation should be a joint decision between the neonatal surgical consultant and the consultant neonatologist.

An anaesthetic review should be encouraged prior to surgery. Echocardiography is helpful in allaying any concerns in this regard if there is any uncertainty. It is also important to ensure that we are prepared to manage any deterioration during surgery. Keeping Nitric Oxide attached and ready to run as well as inotropes ready is best done prior to surgery. Surgery can be performed on HFOV if the above criteria are met.

The following should be ensured prior to surgery

1. Preanaesthetic review
2. Preoperative echo if concerns regarding pulmonary hypertension
3. Consent and discussion with parents
4. FBC and clotting done and results checked
5. Cross-match of 2 units of blood
6. Appropriate central and peripheral access (checked and working)
7. Functioning arterial access
8. Nitric oxide available and run though ready to run
9. There is no evidence of evolving sepsis


Disclaimer: It is your responsibility to check against StaffNet that this printout is the most recent issue of this document

## 5. Guideline-Post operative management

- Standard post operative care to include blood tests (FBC and U&E).
- Obtain a chest X-ray. This helps assess the air pocket and mediastinum.
- Immediately post-operatively, there will be air in the pleural space but this is not usually under tension. There will usually still be some mediastinal shift away from the lesion but this may be less than pre-op. This pleural air can be mistaken for a tension pneumothorax but it rarely needs draining and will be replaced by fluid or the lung will expand into the space over a few days. No needling of the chest should be performed without consultation between a senior surgeon and neonatologist. The instability of the mediastinum can impact upon blood pressure and postoperative PPHN can be challenging to manage. Careful management of blood pressure & echocardiography to evaluate filling and cardiac function is important at this stage.
- Optimising blood pressure, use of Nitric Oxide(even for initial non responders) sedation and analgesia are key in managing postoperative PPHN.
- Inhaled nitric oxide to continue until PPHN resolves. In some cases this can be switched to Sildenafil post extubation.
- These infants may have an increasing ventilator requirement in the immediate postoperative period. Be prepared to escalate ventilation. The same applies for inotropic support. Monitor blood pressure and start inotropes sooner rather than later.
- Need for chest drain or thoracocentesis later on is rare unless there is a large pleural effusion compromising lung function and ventilation. This should be discussed with surgical team where possible.
- Perioperative antibiotics should be as per protocol.
- Enteral feeding should be started as guided by the surgical team. The use of antireflux medication should be guided by the surgeons as Reflux is a common problem.
- Ensure stress for the family is minimized:  Parents should be kept informed of baby's condition and progress. They should be given the opportunity to speak with the surgeon/doctors/nurses.

## 6. Prognosis and Follow Up

The Confidential inquiry into management of CDH has advocated for a family centred approach. The reported survival rates for CDH range from 30 to 80% depending on case selection [29]. This averages out to about 50% in population based studies [30]. The prognosis is significantly worse when other associated congenital anomalies or genetic abnormalities are present. [31] The prognosis for right sided diaphragmatic hernias is poorer as compared to left sided cases. [31,32] **Discussions with the parents about prognosis and of management, and care plans must be carefully documented in the patient record**. Chronic lung disease, ongoing pulmonary hypertension, Neurodevelopmental problems, sensorineural hearing loss and gastroesophageal reflux are common problems in survivors. The need for respiratory support in the NICU and home necessitate involvement of the Paediatric Respiratory team. Ongoing pulmonary hypertension may need involvement of the Paediatric Cardiology team.

Follow up of CDH patients would be organised with the surgical team. Depending on the individual needs of each patient paediatric respiratory, cardiology and Neurodevelopmental follow up may also be needed.
For patients discharged to their local units follow up would be with their local paediatricians and the surgical team. Multidisciplinary input from specialist services might be needed locally.

## 7. Process for Monitoring Compliance/Effectiveness

**Key aspects of the procedural document that will be monitored:**

| What aspects of compliance with the document will be monitored | What will be reviewed to evidence this | How and how often will this be done | Detail sample size (if applicable) | Who will co-ordinate and report findings (1) | Which group or report will receive findings |
|---|---|---|---|---|---|
| **CDH Management Pathway** | **Audit of delivery room managemen t and postnatal care** | **3 years** | | **Consultant or Neonatologist Consultant Paediatric Surgeon** | **Neonatal and Paediatric Surgical consultants** |

# 8. References

1. Field D, Hyman-Taylor P, Bacon C and Draper ES on behalf of MBRRACE-UK. Perinatal Confidential Enquiry– Congenital Diaphragmatic Hernia. Leicester: The Infant Mortality and Morbidity Group, Department of Health Sciences, University of Leicester.2014.

2. Tibboel D, Gaag AV. Etiologic and genetic factors in congenital diaphragmatic hernia. *Clin Perinatol 1996*; 23:689–699.
3. Seetharamaiah R et al. Factors associated with survival in infants with CDH requiring ECMO: a report from the CDH study group. J Pediatr Surg 2009.
4. Soon Ha Yang et al. Reliability of the lung-to-head ratio as a predictor of outcome in foetuses with isolated left congenital diaphragmatic hernia at gestation outside 24-26 weeks. American Journal of Obstetrics and Gynaecology. 2007; 197:30
5. J. Jani et al. Observed to expected lung area to head circumference ratio in the prediction of survival in fetuses with isolated diaphragmatic hernia. Ultrasound in Obstetrics & Gynaecology. 2007; 30:67-71
6. Sbragia L *et al*. Congenital Diaphragmatic Hernia without Herniation of the Liver: Does the Lung-to-Head Ratio Predict Survival? J Ultrasound Med. 2000; 19(12):845-8
7. Harrison MR et al.  A randomised trial of foetal endoscopic tracheal occlusion for severe fetal congenital diaphragmatic hernia. N ENGL J Med 2003; 349: 1916-24
8. J.C Jani et al. Severe diaphragmatic hernia treated by foetal endoscopic tracheal occlusion. Ultrasound in Obstetrics & Gynecology. 2009; 34:304-310

9. Philip DeKoninck et al. Results of Foetal Endoscopic Tracheal Occlusion for congenital diaphragmatic hernia and the set up of the randomized controlled TOTAL trial. Early Human development. 2011; 87:619-624

10. George Graham et al. Antenatal Diagnosis of Congenital Diaphragmatic Hernia. Seminars in Perinatology . Volume 29, Issue 2, April 2005, Pages 69-76

11. Wright JCE, Budd JLS, Field DJ, Draper ES. Epidemiology and outcome of congenital diaphragmatic hernia: a 9-year experience. Paediatric and Perinatal Epidemiology 2010;25: 144

12. Reiss I, Schaible T, van der Hout L et al. Standardized postnatal management of infants with congenital diaphragmatic hernia in Europe: the CDH EURO consortium consensus. Neonatology 2010; 98: 354.

13. Bos AP, Hussain SM et al. Radiographic evidence of bronchopulmonary dysplasia in high-risk congenital diaphragmatic hernia survivors. Paediatric Pulmonology. 1993; 15:231-234

14. Logan JW, Cotten CM et al. Mechanical ventilation strategies in the management of congenital diaphragmatic hernia. Semin Pediatr Surg. 2007; 16:115-125

15. Sakurai Y. Pulmonary barotrauma in congenital diaphragmatic hernia: A clinicopathological correlation. J Pediatr Surg 1999, 34:1813.

16. Vanamo K, Rintala R, Sivijarvi A, et al: Long-term pulmonary sequelae in survivors of congenital diaphragmatic defects. J Pediatr Surg 1996; 31:1096

17. Boloker J, Bateman DA, Wung JT, Stolar CJ: Congenital diaphragmatic hernia in 120 infants treated consecutively with permissive hypercapnia/spontaneous respiration/elective repair. J Paediatr Surg 2002; 37: 357

18. Frenckner B, Ehrén H, Granholm T et al. Improved results in patients who have congenital diaphragmatic hernia using preoperative stabilization, extracorporeal membrane oxygenation, and delayed surgery. J Pediatr Surg 1997; 32:1185
19. Kays DW, Langham MR Jr, Ledbetter DJ, Talbert JL: Detrimental effects of standard medical therapy in congenital diaphragmatic hernia. Ann Surg 1999; 230:340
20. Wung JT, Sahni R, Moffitt ST et al: Congenital diaphragmatic hernia: survival treated with very delayed surgery, spontaneous respiration and no chest tube. J Pediatr Surg 1995; 30:406
21. Lieke van den Hout et al. The VICI-trial: high frequency oscillation versus conventional mechanical ventilation in newborns with congenital diaphragmatic hernia: an international multicentre randomized controlled trial. BMC Paediatrics. Volume 11.
22. Bhuta T, Clark RH, Henderson-Smart DJ: Rescue high frequency oscillatory ventilation vs conventional ventilation for infants with severe pulmonary dysfunction born at or near term. Cochrane Database Syst Rev 2001:CD002974.
23. Finner NN, Barrington KJ: Nitric oxide for respiratory failure in infants born at or near term. Cochrane database Syst Rev 2006
24. Inhaled nitric oxide and hypoxic respiratory failure in infants with congenital diaphragmatic hernia. The Neonatal Inhaled Nitric Oxide Study Group (NINOS). Pediatrics 1997; 99:838
25. Langham MR Jr, Krummel TM, Bartlett RH, et al: Mortality with extracorporeal membrane oxygenation following repair of congenital diaphragmatic hernia in 93 infants. J Pediatr Surg 1987; 22:1150


Disclaimer: It is your responsibility to check against StaffNet that this printout is the most recent issue of this document

26. Van Meurs KP, Newman KD, Anderson KD et al: Effect of extracorporeal membrane on survival of infants with congenital diaphragmatic hernia. J Pediatr 1990; 117:954
27. I. Sluiter, C.P. van de Ven et al. Congenital diaphragmatic hernia: still a moving target. Seminars in Fetal and Neonatal Medicine. 2011; 16:139-144
28. Extracorporeal Life Support Organisation registry. ELCS registry report. Ann Arbor, MI: ELSO; July 2010
29. Mara B. Antonoff, Virginia A. Hustead et al: Protocolised management of infants with congenital diaphragmatic hernia: effect on survival. J Pediatr Surg 2011; 46:39-46
30. Cools F, Offringa M: Neuromuscular paralysis for newborn infants receiving mechanical ventilation. Cochrane database Syst Rev 2005:CD002773
31. Tovar A:  Orphanet Journal of Rare Diseases 2012; 7:1
32. Bagolan P, Morini F: Long-term follow up of infants with congenital diaphragmatic hernia. Seminars in Pediatric Surgery 2007; 16: 134.

# Appendix 1A: Equipment for Airway & Access in Delivery Suite

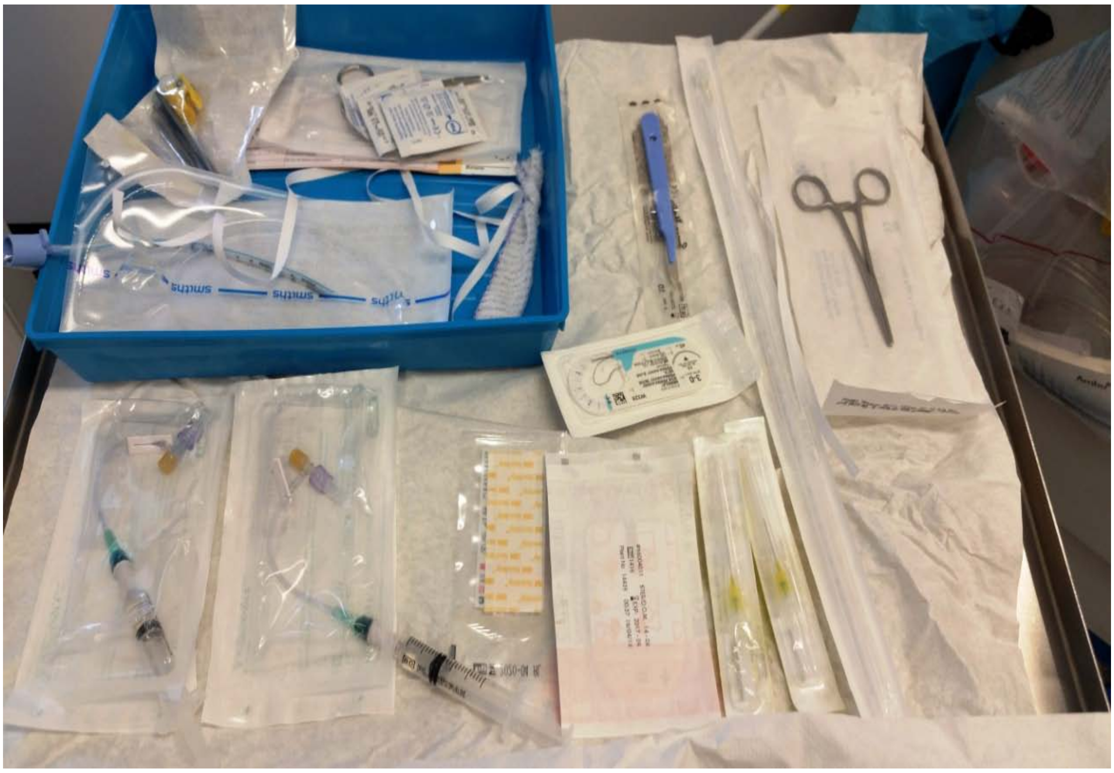

- Pre-cut endotracheal tubes with introducers
- Net-elast hat and ties
- Laryngoscope and blades
- Suction catheter including a Yankuer sucker
- 24G Cannulas
- Single lumen UVC with cord tie and blade (For failure of cannulation)
- T- piece flushed through and attached to syringe
- Aliquots of normal saline 10mls/kg (depending on estimated weight of baby)
- Nasogastric tube with purple syringes


Disclaimer: It is your responsibility to check against StaffNet that this printout is the most recent issue of this document

## Appendix 1B: Drugs for Sedation and Paralysis

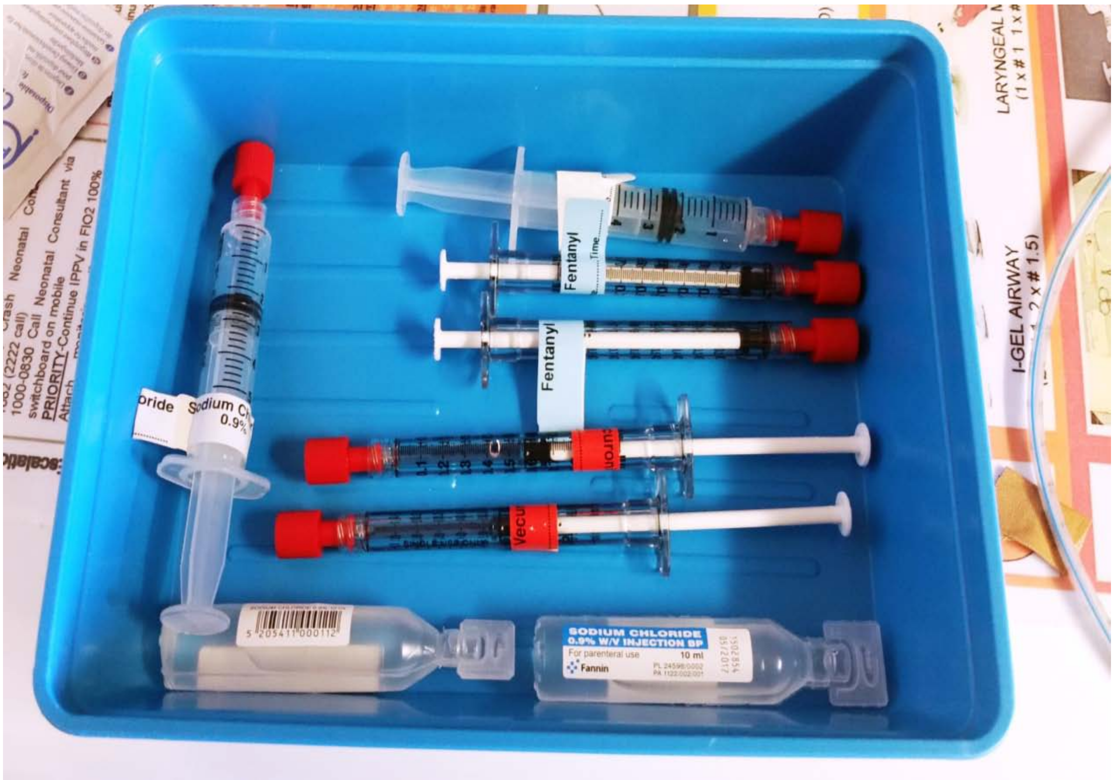


- <u>Medications</u>
- 2 doses of sedation, preferably Fentanyl. (4 microgram/kg)
- 2 doses of muscle relaxant, also prescribed according to estimated weight
- 0.9% saline flushes, two 5ml syringes as flush
- If no IV access is obtainable and paralysis is required Suxamethonium can be given at a dose of 4mg/kg

## Appendix 2- Imminent Delivery of a baby with CDH (Preparation s key!!!)

### Human Factors

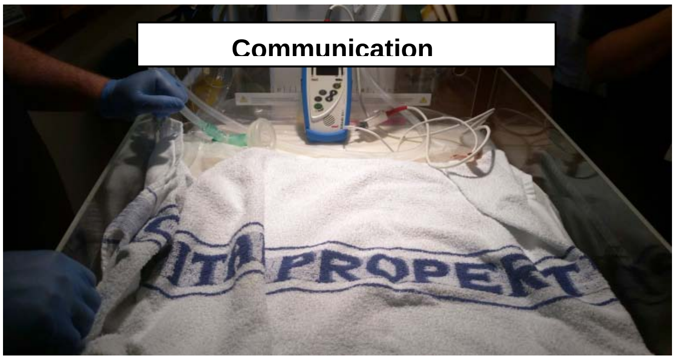


**Prebrief**

**Who is team leader?**

**Delegate who is doing ABC, checking the equipment and the resuscitaire**

**Is a consultant on the way if not already there?**

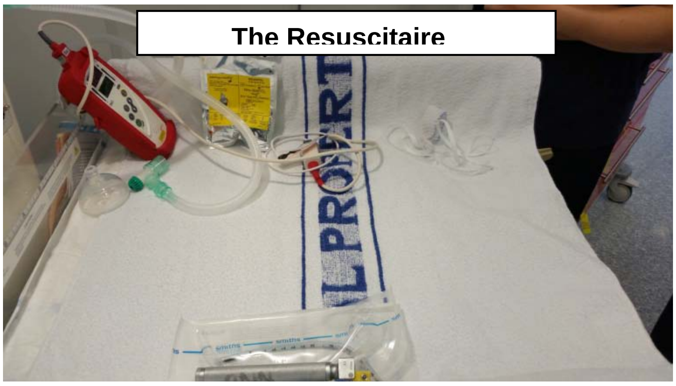


**Have you set PIP and PEEP?**

**Does it have oxygen/enough oxygen?**

**Does your laryngoscope work?**

**Do you have capnography?**

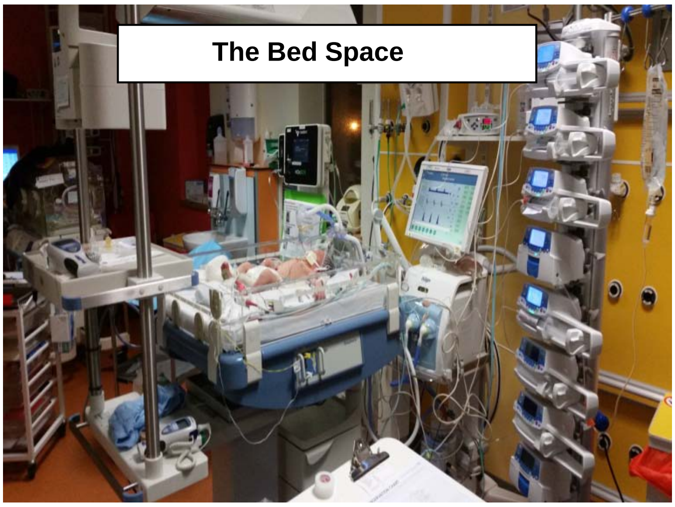


**Is a bed space with the appropriate ventilator and equipment ready?**

**Has the Nitric Oxide checked and run through?**

**Is a Sensormedics available?**

### Is the PICU consultant/ PICU aware?

# Appendix 3: CDH Management Pathway

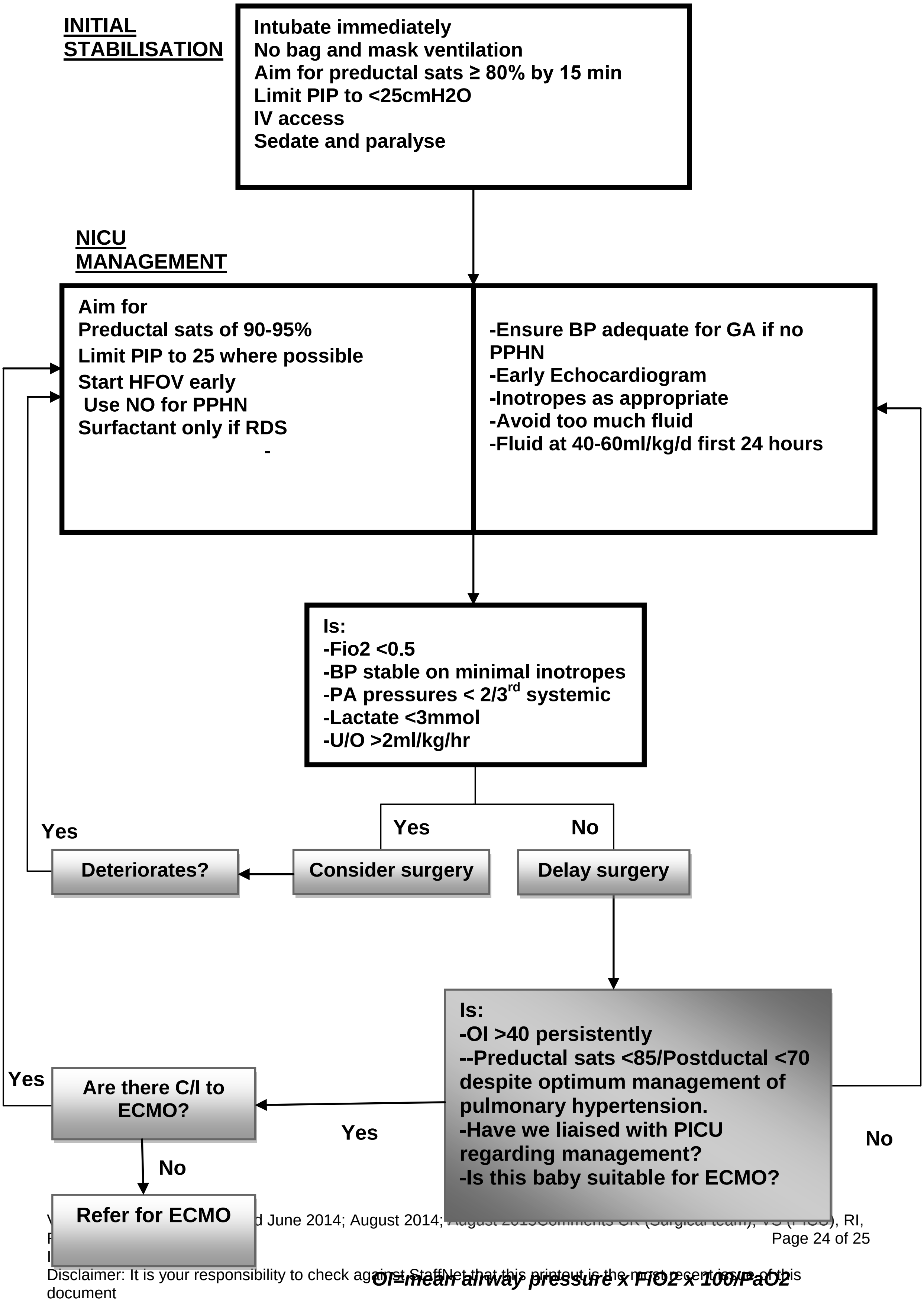



Disclaimer: It is your responsibility to check against StaffNet that this printout is the most recent issue of this document

| **Document Monitoring Information** | |
|---|---|
| **Approval Committee:** | **Neonatal Governance** |
| **Date of Approval:** | **Insert Date** |
| **Ratification Committee:** | **Insert Name of Committee (Policy Ratification and Monitoring Group (PRAMG) for Level 1 documents)** |
| **Date of Ratification:** | **Insert Date** |
| **Signature of ratifying Committee Group/Chair:** | **Insert Signature or name (Chair of PRAMG if Level 1 document)** |
| **Lead Name and Job Title of originator/author or responsible committee/individual:** | **Insert name and job title of responsible individual/author or responsible committee** |
| **Policy Monitoring (Section 6) Completion Date:** | **Insert Date** |
| **Policy Monitoring to be presented to responsible committee or PRAMG:** | **Insert Date or Month applicable** |
| **Target audience:** | **Neonatal, PICU, Maternity, Paediatric Surgery** |
| **Key words:** | |
| **Main areas affected:** | **Trust wide for Level 1 documents** |
| **Summary of most recent changes if applicable:** | **None** |
| **Consultation:** | **As stated in the footer** |
| **Equality Impact Assessment completion date:** | |
| **Number of pages:** | **25** |
| **Type of document:** | **Guideline** |
| **Does this document replace or revise an existing document** | **No** |
| **Should this document be made available on the public website?** | **No** |
| **Is this document to be published in any other format?** | **No** |

**The Trust strives to ensure equality of opportunity for all, both as a major employer and as a provider of health care. This document has therefore been equality impact assessed to ensure fairness and consistency for all those covered by it, regardless of their individual differences, and the results are available on request.**
